# Acceptability of Iron- and Zinc-Biofortified Pearl Millet (ICTP-8203)-Based Complementary Foods among Children in an Urban Slum of Mumbai, India

**DOI:** 10.3389/fnut.2017.00039

**Published:** 2017-08-25

**Authors:** Samantha Lee Huey, Sudha Venkatramanan, Shobha A. Udipi, Julia Leigh Finkelstein, Padmini Ghugre, Jere Douglas Haas, Varsha Thakker, Aparna Thorat, Ashwini Salvi, Anura V. Kurpad, Saurabh Mehta

**Affiliations:** ^1^Division of Nutritional Sciences, Cornell University, Ithaca, NY, United States; ^2^Department of Food Science and Nutrition, SNDT Women’s University, Mumbai, India; ^3^St. John’ Research Institute, Division of Nutrition, Bangalore, India; ^4^Institute for Nutritional Sciences, Global Health, and Technology (INSiGHT), Cornell University, Ithaca, NY, United States

**Keywords:** pearl millet, ICTP-8203, iron, iron deficiency, biofortification, acceptability, children

## Abstract

Biofortification, a method for increasing micronutrient content of staple crops, is a promising strategy for combating major global health problems, such as iron and zinc deficiency. We examined the acceptability of recipes prepared using iron- and zinc-biofortified pearl millet (FeZnPM) (~80 ppm Fe, ~34 ppm Zn, varietal ICTP-8203), compared to conventional pearl millet (CPM) (~20 ppm Fe, ~19 ppm Zn) in preparation for an efficacy trial. Our objective was to examine the acceptability of FeZnPM compared to CPM among young children and mothers living in the urban slums of Mumbai. Standardized traditional feeding program recipes (*n* = 18) were prepared with either FeZnPM or CPM flour. The weight (g) of each food product was measured before and after consumption by children (*n* = 125) and the average grams consumed over a 3-day period were recorded. Mothers (*n* = 60) rated recipes using a 9-point hedonic scale. Mean intakes and hedonic scores of each food product were compared using *t*-tests across the two types of pearl millet. There were no statistically significant differences in consumption by children (FeZnPM: 25.27 ± 13.0 g; CPM: 21.72 ± 6.90 g) across the food products (*P* = 0.28). Overall mean hedonic scores for all recipes were between 7 to 9 points. CPM products were rated higher overall (8.22 ± 0.28) compared to FeZnPM products (7.95 ± 0.35) (*P* = 0.01). FeZnPM and CPM were similarly consumed and had high hedonic scores, demonstrating high acceptability in this population. These results support using these varieties of pearl millet in a proposed trial [http://Clinicaltrials.gov ID: NCT02233764; Clinical Trials Registry of India (CTRI), reference number REF/2014/10/007731, CTRI number CTRI/2015/11/006376] testing the efficacy of FeZnPM for improving iron status and growth.

## Introduction

Iron deficiency is the most common micronutrient deficiency in the world and particularly impacts child health in resource-limited settings such as urban slums in India (1). With or without anemia, low iron status impairs growth (2), immune (3), and cognitive functions (4) in infants and young children. Preliminary data from children in our study population in Mumbai showed that iron intake was 35% that of the Indian RDA (5, 6) and that nearly 60% of children were iron deficient (serum ferritin < 12 ng/mL) (7). Zinc deficiency affects nearly 2 billion people worldwide and is associated with short stature, delayed development, and increased morbidity particularly from diarrhea (8). Collectively, these deficits, particularly during the first 1,000 days of life, result in long-term health consequences such as poor school performance and decreased work capacity (9).

Biofortification of staple crops with iron is a sustainable agricultural approach that can help prevent and treat iron deficiency in vulnerable populations (10). In the process of biofortification, the concentration and bioavailability of essential micronutrients in staple food crops is increased by traditional plant breeding techniques (11, 12). Pearl millet (*Pennisetum glaucum*, and locally known as *bajra* in Hindi) is a nutritious, hardy, drought-tolerant grain that is a staple of the traditional diet in many areas of India including Maharashtra, Gujarat, and Rajasthan, where per-capita consumption is 80–100 g (10, 13).

A locally grown variety of iron- and zinc-biofortified pearl millet (ICTP-8203 or FeZnPM) with four times higher iron concentration than conventional pearl millet (CPM) has the potential to improve iron status in these populations (10, 14). Traditionally, pearl millet is ground into flour, roasted, and consumed in the form of non-leavened breads called *bhakri* or *roti* as part of the daily diet. ICTP-8203, shown to have comparable if not higher yield than conventional varieties (15), has demonstrated efficacy in improving iron status in older children who consumed *bhakri* twice daily for 6 months (10, 16). However, flatbreads like *bhakri* are not ideal weaning or complementary foods for young children and infants to consume due to their tougher and chewier texture. The main objective of this study was to formulate and test the acceptability (in terms of volume consumed and sensory characteristics) of new pearl millet-based palatable complementary food products for weaning infants. The food products with highest acceptability would be ideal candidates for a randomized controlled trial testing the efficacy of biofortified pearl millet for improving iron status in infants and young children.

## Materials and Methods

### Intervention

Pearl millets CPM and ICTP-8203 were transported from Maharashtra and Gujarat, respectively, in 50-kg gunny bags and stored in a climate-controlled space (humidity level < 50%, 25°C). Pearl millet was transferred from the gunny bags into steel canisters and transported to SNDT Women’s University, Mumbai, India, for storage and preparation. The concentrations of iron, zinc, and aluminum in whole grain were quantified after nitric acid digestion using inductively coupled plasma optical emission spectrometry (ICP-OES) at the International Crops Research Institute for the Semi-Arid Tropics research laboratory in Telangana, India. Confirmatory testing in the USDA-ARS lab at Cornell University (courtesy, Dr. Ray Glahn), was used to test for iron and zinc concentrations, along with assessment of potential contamination *via* aluminum concentrations using ICP-OES.

### Recipe Formulation and Production

Ingredients and formulations were based on traditional weaning and complementary foods in this region. The teaching food laboratory at SNDT Women’s University was utilized for recipe development. The recipes were prepared by Master of Science in Food, Science, and Nutrition students and staff from the department of Food Science and Nutrition under the guidance of the study investigators and community coordinator. Standard hygiene and sanitation procedures were maintained throughout.

### Study Setting

The study site was a feeding center within a large slum known locally as Nehru Nagar, in Vile Parle, a suburb in Mumbai. The feeding center was a room generally used for Integrated Child Development Services activities (17). This room was equipped with electricity and fans.

### Study Design and Protocol

This study was conducted from January to December 2015. Mothers were given an information sheet describing the pearl millet crop and its potential health benefits. Community health workers explained to mothers that they would be given foods prepared using both high-iron/zinc and conventional pearl millets to consume.

Based on preliminary data indicating preferred times of day for feeding, mothers were requested to come to the feeding center between 11:00 a.m. and 5:00 p.m. each day to maximize participation. If a child refused food or if mothers needed to leave before the child finished, mothers were requested to return to the center to continue feeding. Mothers were also encouraged to come between breast-feeding periods to encourage consumption of the complementary food. Participants consumed each food product over a 3-day period to allow the child to become familiar with any potential new tastes or flavors. First, all CPM products were tested for acceptability (days 1–3), followed by all FeZnPM products (days 4–6).

Research assistants weighed each food product before and after feeding to the nearest 0.01 g to determine the net number of grams consumed of each food product. Both research assistants and community health workers assisted mothers with feeding, if needed. The proportion of pearl millet consumed in each food product was then back-calculated *via* the proportion of pearl millet initially used to prepare the given recipe.

Concurrently with their infants, mothers were also given a sample of each food on the day of its introduction and were asked to rate its acceptability using a 9-point hedonic scale while a research assistant assisted with feeding their child. The hedonic score was based on a 9-point scale (1 = lowest rating, “dislike extremely”; 9 = highest rating, “like extremely”) similar to scales used in previous studies of consumer acceptance and assessed four parameters: taste, smell, color, and overall acceptability (18–20).

### Anthropometry

Mothers of *N* = 38 children consented to anthropometric measurements. Recumbent length (centimeters) (Meditrin Infantometer, Mumbai, India), weight (kilograms) (Meditrin scale model 385, Mumbai, India), and mid-upper-arm circumference (centimeters) (Hardik Meditech model #HM009, New Delhi, India) were measured by trained study personnel.

### Ethical Approval

Intersystems Biomedica Ethics Committee, Mumbai, India, approved the study protocol (ISBEC/NR-17/KM-JVJ/2014). The nature of the study was explained to the mothers and written informed consent was obtained from the mothers or the caregivers. All participants gave written informed consent.

### Data Analysis

Two outcome variables in this study were used: the average net number of grams of pearl millet consumed in each food product among children and the mean hedonic score for each food product among mothers. To compare the mean intake of each pearl millet variety as a proportion of the total food product, as well as mean hedonic scores, data were first tested for normality. Because data were not normally distributed, results from the non-parametric Hodges–Lehmann–Sen test were compared with results from two-sample *t*-tests. Results were similar, thus we report means and *t*-tests here for comparability with other publications. Anthropometric *z*-scores [weight-for-age (WAZ), length-for-age (LAZ), and weight-for-length (WLZ)] were calculated using World Health Organization guidelines; underweight, stunting, and wasting were defined as WAZ < −2, LAZ < −2, and WLZ < −2 SDs from the WHO reference standard, respectively (21). SAS version 9.4 (SAS Institute, Cary, NC, USA) was used for all analysis.

## Results

### Development of Pearl Millet Products

A total of 18 food products were tested in an open cohort of 125 children aged 6–24 months living in Nehru Nagar, Vile Parle, Mumbai. These food products included freshly prepared and shelf stable foods, both sweet (#1–8) and savory (#9–18) recipes (Table 1). We formulated recipes appropriate to various stages of development, i.e., a softer porridge-like consistency food product (*khichdi*) for very young children and more solid foods such as *pakoda* and *thepla* for older children. Iron and zinc concentration in the biofortified pearl millet was 82.74 and 34.17 ppm, respectively; in the conventional pearl millet, iron and zinc concentration were 21.24 and 19.34 ppm, respectively, and aluminum concentrations indicated low levels of contamination (<10 ppm per varietal).

**Table 1:** Recipe description and methodology.

No.	Sweet food product (% pearl millet)		Description and protocol
	Iron/zinc content (mg)/100 g		
1	Cookies (53%)		Soft, sweet biscuits
	Yield: 470 g (40 pieces)		Ingredients: pearl millet flour (250 g), powdered sugar (125 g), ghee (83 g), cocoa powder (12.5 g), and baking powder (2.5 g)
	Serving size: 2 pieces		
	**FeZnPM**	**CPM**	Directions: preheat oven to 120°C (248 F). Sift pearl millet flour, cocoa powder, and baking powder into a bowl and set aside. Cream sugar and ghee. Combine the flour mixture with the creamed ghee and sugar and mix well. Roll out, cut into squares, and bake for 10–12 min. Let cool and serve
	Fe: 5.23	Fe: 1.83	
	Zn: 1.70	Zn: 0.99	

2	Peanut *Laddu* (46%)		Sweet peanut-flavored bite-sized snacks
	Yield: 300 g (15 *laddu)*		Ingredients: pearl millet flour (150 g), powdered sugar (100 g), ghee (50 g), roasted and ground peanuts (25 g)
	Serving size: 2 *laddu*		
	**FeZnPM**	**CPM**	Directions: melt ghee in a non-stick pan and add pearl millet flour; roast until golden brown and remove from heat. Add peanuts, mix, then add sugar and cardamom and combine. Roll into 1″ balls and allow to cool. Store in a sealed container in the fridge. Serve at room temperature
	Fe: 4.19	Fe: 1.74	
	Zn: 1.40	Zn: 1.04	

3	*Sheera* (56%)		Sweet porridge/pudding
	Yield: 450 g		Ingredients: pearl millet flour (150 g), powdered sugar (100 g), ghee (50 g), ground cardamom (3 g), and boiled water (250 mL)
	Serving size: 50 g		
	**FeZnPM**	**CPM**	Directions: melt ghee in a non-stick pan and add pearl millet flour; roast until golden brown and remove from heat. Add sugar and mix, then add boiled water and stir to combine. Add cardamom and cook 5–10 min. Serve warm or at room temperature
	Fe: 5.28	Fe: 1.34	
	Zn: 1.53	Zn: 0.89	

4	*Churma laddu* (33%)		Traditional spiced sweet balls
	Yield: 320 g (15 laddu)		Ingredients: pearl millet flour (150 g), *jaggery* (120 g), ghee (22 g), ground cardamom (3 g), and water (15 mL)
	Serving size: 2 *laddu*		
	**FeZnPM**	**CPM**	Directions: sift flour and combine with water; knead until soft. Form into small round flatbreads (*bhakri*) using additional pearl millet flour as needed, roast both sides, and let cool. Grind *bhakri* into a powder. Heat ghee and add jaggery; once melted, combine with powdered *bhakri*. Form into 1″ balls and allow to cool. Serve
	Fe: 4.33	Fe: 1.36	
	Zn: 1.17	Zn: 0.88	

5	Cake (21%)		Soft sweet bread
	Yield: 1,150 g (23 slices)		Ingredients: pearl millet flour (250 g), refined wheat flour (125 g), powdered sugar (250 g), condensed milk (31 g), baking soda (9.3 g), butter (219 g), milk (269 mL), and cocoa powder (12.5 g)
	Serving size: 1 slice		
	**FeZnPM**	**CPM**	Directions: preheat oven to 120°C (248 F). Sift pearl millet flour and wheat flour into a bowl. Combine with baking soda and cocoa powder and set aside. Cream sugar and butter; add condensed milk and mix well with flour mixture. Bake 10–15 min or until a knife comes out clean. Let cool, slice, and serve
	Fe: 2.47	Fe: 1.42	
	Zn: 0.83	Zn: 0.80	

6	*Nankhatai* (49%)		Indian shortbread
	Yield: 700 g (60 pieces)		Ingredients: pearl millet flour (335 g), sugar (176 g), ghee (176 g), and ground cardamom (2 g)
	Serving size: 2 pieces		
	**FeZnPM**	**CPM**	Directions: preheat oven to 120°C (248 F). Sift pearl millet flour and cardamom into a bowl and set aside. Cream sugar and ghee. Combine the flour mixture with the creamed ghee and sugar and mix well. Form 1″ balls and press to flatten. Bake for 10–12 min. Let cool and serve
	Fe: 5.12	Fe: 1.34	
	Zn: 1.30	Zn: 0.82	

7	*Porridge* (61%)		Sweet pudding
	Yield: 1,700 g		Ingredients: coarsely ground pearl millet (500 g), powdered sugar (350 g), boiling water (850 mL), and ground cardamom (1.5 g)
	Serving size: 50 g		
	**FeZnPM**	**CPM**	Directions: add pearl millet to boiling water and stir. Add sugar and cardamom; cook until the pearl millet has absorbed the water and a smooth consistency is reached. Serve warm
	No data available		

8	*Puranpoli* (52%)		Flatbread with sweet filling
	Yield: 1,260 g (25 pieces)		Ingredients: pearl millet flour (300 g), coarsely ground pearl millet (200 g), water (300 mL), *jaggery* (200 g), refined wheat flour (50 g), water (192 mL), ghee (20 g), and cardamom powder (1.5 g)
	Serving size: 1 piece		
	**FeZnPM**	**CPM**	Directions: for *puran*, combine coarsely grouned pearl millet flour and 192 mL water; cook using a pressure cooker. Add *jaggery* and stir to break up any lumps; add cardamom powder. For *poli* dough, combine pearl millet flour, wheat flour and 300 mL water and make equal sized balls of dough 3″ in diameter. Flatten each ball into a circular shape (*roti*) using a rolling pin. On half of the *roti*, spread an even layer of *puran*. Place another *roti* on top and pinch to seal the edges. Roast the *puranpoli* on both sides. Once cooked through, brush ghee on top and serve warm
	No data available		

	**Savory food product (% pearl millet)**		**Description and protocol**
	**Iron/zinc content (mg)/100 g**		

9	*Khichdi* (55%)		Savory porridge with lentils
	Yield: 1,000 g		Ingredients: pearl millet flour (300 g), moong beans (150 g), ghee (75 g), asafoetida (5 g), ground turmeric (6 g), cumin seeds (12 g), and water (450 mL)
	Serving size: 50 g		
	**FeZnPM**	**CPM**	Directions: roast pearl millet flour in ghee until golden brown. Add water and rest of the ingredients; cook on a medium flame until soft and thickened. Thin with water if needed. Serve warm
	No data available		

10	*Upma* (71%)		Thick, soft dry porridge
	Yield: 3,000 g		Ingredients: coarsely ground pearl millet (600 g), grated carrot (120 g), chopped tomato (87 g), cilantro (5 g), ground turmeric (15 g), green chili paste (3 g), salt (20 g), and water (2,100 mL)
	Serving size: 50 g		
	**FeZnPM**	**CPM**	Directions: roast pearl millet in ghee on a medium flame until golden brown. Add carrot and tomato; cook until vegetables are soft. Add hot water and stir continuously, then add spices and green chili paste and cook until water has evaporated and *upma* has reached a soft consistency. Thin with water as needed. Serve warm.
	No data available		

11	*Dhokla* (54%)		Steamed rice cake with tempered sauce, east Indian recipe
	Yield: 365 g (23 pieces)		Ingredients: pearl millet flour (75 g) + coarsely ground pearl millet flour (75 g), semolina (38 g), plain yogurt (55 g), baking soda (2.5 g), ground turmeric (2.5 g), sugar (2.5 g), salt (2.5 g), vegetable oil (35 mL), water (130 mL), mustard seeds (2 g), cumin seeds (2 g), asafoetida (1 g), curry leaves (3 g), and cilantro (3 g)
	Serving size: 2 pieces		
	**FeZnPM**	**CPM**	Directions: combine pearl millet flours, semolina, and yogurt; rest overnight at room temperature to ferment. After fermentation, add remaining ingredients (baking soda, ground turmeric, sugar, salt, 25 mL vegetable oil, and water) and mix well. Pour batter into steamer and steam for 15 min on medium flame until a knife/toothpick comes out clean. Remove *dhokla* from steamer and allow to cool for 5 min. Cut into squares or diamonds. Next, heat remaining 10 mL vegetable oil and add remaining ingredients (except cilantro) until the mustard seeds begin to crackle. Pour this mixture over the *dhokla* and toss gently; garnish with cilantro
	Fe: 6.66	Fe: 1.24	
	Zn: 1.80	Zn: 0.82	

12	*Idli* (56%)		Steamed rice cake, south Indian recipe
	Yield: 455 g (11 pieces)		Ingredients: coarsely ground pearl millet (150 g), moong beans (108 g), fenugreek seeds (5 g), salt (2.5 g), baking soda (2.5 g), water (30 mL), and oil (for greasing the steamer pan)
	Serving size: 2 pieces		
	**FeZnPM**	**CPM**	Directions: wash and soak pearl millet and moong beans; set aside for 5 h. Add fenugreek seeds and soak with pearl millet/moong bean mixture. Drain off the extra water from this mixture and add salt; blend in a blender until smooth, adding cold water as necessary. Allow to ferment at room temperature for 8 h. Gently mix (do not overmix). Heat water on medium-high heat. Grease *idli* pan with oil. If needed, add water to reach desired consistency. Pour batter into each mold and steam *idli* for 15 min on medium-high heat. Remove from *idli* pans and allow to cool for 2–3 min. Loosen each *idli* from the pans and serve with chutney
	Fe: 7.42	Fe: 2.65	
	Zn: 1.80	Zn: 1.58	

13	Vegetable cutlet (78%)		Pan-fried pearl millet and vegetable patties
	Yield: 300 g (6 pieces)		Ingredients: coarsely ground pearl millet (150 g), sliced carrot (20 g), green chilies (2.5 g), cilantro leaves (2.5 g), asafoetida (2.5 g), ground turmeric (2.5 g), salt (5 g), vegetable oil (6.5 mL), and water (150 mL)
	Serving size: 2 pieces		
	**FeZnPM**	**CPM**	Directions: pressure cook pearl millet for 1–2 whistles on a medium flame. Mash pearl millet until soft. Add remaining ingredients and mix well. Form patties, 2″ in diameter. Pan fry each patty on both sides until golden brown. Serve with ketchup
	Fe: 8.56	Fe: 1.78	
	Zn: 1.29	Zn: 0.64	

14	*Kothimbir wadi* (76%)		Pan-fried soft patties with cilantro
	Yield: 295 g (32 pieces)		Ingredients: pearl millet flour (150 g), finely chopped cilantro (2.5 g), green chilies (2.5 g), ground ginger (2.5 g), sesame seeds (2.5 g), ground cumin (2.5 g), ground turmeric (2.5 g), salt (2.5 g), vegetable oil (30 mL), and water (102 mL)
	Serving size: 2 pieces		
	**FeZnPM**	**CPM**	Directions: in a bowl, combine pearl millet and cilantro. Add spices and sesame seeds, and knead into a firm dough. Make equal sized rolls and steam for 10–15 min. Allow to cool. Cut into equal pieces and pan fry on low heat in 2 tsp of oil. Serve with green chutney or tomato sauce
	Fe: 8.29	Fe: 5.59	
	Zn: 3.01	Zn: 1.17	

15	*Thepla* (65%)		Flatbread with fenugreek leaves
	Yield: 1,300 g (52 pieces)		Ingredients: pearl millet flour (500 g), water (600 mL), plain yogurt (134 g), ginger (6.6 g), garlic (6.6 g), sesame seeds (6.6 g), ground cumin (3.3 g), fenugreek leaves (6.6 g), green chilies (2.6 g), salt (10 g), oil (25 mL), and water (as needed)
	Serving size: 1 piece		
	**FeZnPM**	**CPM**	Directions: combine all ingredients to form a soft dough, adding water as needed. Make equal sized balls and use a rolling pin to make thin flat breads. Fry both sides using oil until cooked thoroughly. Serve warm or at room temperature
	

16	*Pav* (40%)		Bread (*pav*) with pureed vegetables (*bhaji*)
	Yield: 305 g		*Pav ingredients*: pearl millet flour (150 g), refined wheat flour (25 g), milk (150 mL), ghee (50 g), baking soda (2.5 g), and butter (as needed)
	Serving size: 50 g		
	**FeZnPM**	**CPM**	Directions: preheat oven to 100°C (212 F). Sift pearl millet flour, wheat flour, and baking soda together. In another bowl, combine ghee, milk powder, and sifted pearl millet flour. Add to milk and mix. Pour the batter into a greased baking tray. Bake for 10–12 min. Let cool and cut into square pieces. Serve with pearl millet *bhaji*
	Fe: 5.59	Fe: 1.74	
	Zn: 1.81	Zn: 1.11	

			Bhaji ingredients: coarsely ground pearl millet (150 g), onion (30 g), tomato powder (30 g), green bell pepper (25 g), green chilies (2.5 g), chopped cilantro (2.5 g), pav bhaji spice mix (7.5 g), vegetable oil (30 mL), chili powder (2.5 g), ground turmeric (3 g), salt (7.5 g), and water (490 mL)
	*Bhaji* (51%)		
	Yield: 630 g		
	Serving size: 50 g		Directions: pressure cook pearl millet flour for 1–2 whistles on a medium flame. Once cooked, mash the pearl millet. Separately grind two pastes: (1) onion and green pepper, and (2) chilies and garlic. In a pan, heat oil and add onion/pepper paste. Sautée until the onion becomes translucent and then add tomato powder, turmeric, salt, and *pav bhaji* masala powder. Mix well and fry on medium heat for 2–3 min. Add the cooked pearl millet and mix well with the masala. Add water as needed and stir until desired consistency is reached, then add cilantro. Serve warm with pearl millet *pav*
	**FeZnPM**	**CPM**	
	Fe: 6.71	Fe: 1.72	
	Zn: 0.73	Zn: 0.35	

17	*Pakoda* (58%)		Deep fried pearl millet fritters
	Yield: 275 g		Ingredients: pearl millet flour (150 g), plain yogurt (60 g), vegetable oil (35 mL), garlic paste (5 g), ground cumin (2.5 g), salt (2.5 g), ground turmeric (2.5 g), and water (118 mL)
	Serving size: 50 g		
	**FeZnPM**	**CPM**	Directions: in a bowl, mix pearl millet flour, salt, yogurt, cumin, garlic paste, turmeric, and 1 tsp hot oil. Add water and stir well. Heat oil in a large *kadai* (pan). If a small amount of batter dropped into the hot oil rises to the surface immediately, the oil is ready for frying the pakoda. Reduce the heat to medium, take a spoonful of batter and drop into the oil; fry until golden brown on all sides. Remove from oil and drain on paper towels. Serve warm, with ketchup
	Fe: 6.60	Fe: 2.43	
	Zn: 2.14	Zn: 1.28	

18	*Vada* (42%)		Deep fried spicy pearl millet patties
	Yield: 400 g (11 pieces)		Ingredients: coarsely ground pearl millet (150 g), pearl millet flour (50 g), green chilies (2.5 g), salt (5 g), ground ginger (3 g), ground turmeric (3 g), cilantro (1.5 g), ground cumin (2.5 g), salt (2.5 g), asafoetida (1.5 g), and water (395 mL), vegetable oil (for frying)
	Serving size: 2 pieces		
	**FeZnPM**	**CPM**	Directions: for *vada*, rinse coarse pearl millet with water and pressure cook. Let cool, then mash until soft. Add 2.5 g salt, ginger, 1.5 g turmeric, cilantro, and 300 water; mix well, form 2″ balls and set aside. In another bowl, mix pearl millet flour with 2.5 g turmeric, cumin, remaining 2.5 g salt, asafoetida, and 1 tsp of hot oil. Add ~95 mL water if necessary to create a thin consistency. Heat oil in a large *kadai* (pan). Dip each *vada* ball into the batter and deep fry until golden brown. Allow to cool. Serve with chutney or ketchup
	Fe: 5.27	Fe: 1.18	
	Zn: 1.22	Zn: 0.64	

### Acceptability: Infants and Mothers

Population characteristics of an *N* = 38 subset of children may be found in Table 2. No statistically significant differences in infants’ overall mean consumption of foods developed from ICTP-8203-Fe PM (mean ± SD; 25.3 ± 13.0 g), were found, compared to the conventional pearl millet (21.7 ± 6.9 g) (*P* = 0.28) (Table 3). There was no difference in mean grams consumed from either sweet or savory food products in the CPM (*P* = 0.1) and FeZnPM (*P* = 0.6) groups. FeZnPM products with the highest acceptability among children included *churma laddu* (31.5 g), vegetable cutlet (53.2 g), and *dhokla* (28.1 g), while the least-consumed FeZnPM food products included *upma* (9.3 g), *khichdi* (9.8 g), and *pav* (14.4 g).

**Table 2:** Characteristics of participants at enrollment.

Characteristic	*n*/*N* (%) or mean ± SD
Sex female	14/37 (37.8)
Age (months)	15.9 ± 4.6
Weight (kg)	8.62 ± 1.23
Length (cm)	73.04 ± 5.10
Mid-upper arm circumference (cm)	13.81 ± 0.98
Weight-for-length *Z*-score	−0.41 ± 0.91
Length-for-age *Z*-score	−2.07 ± 1.37
Weight-for-age *Z*-score	−1.44 ± 1.11
Stunting (LAZ < −2)	16/36 (44.4)
Underweight (WAZ < −2)	10/36 (27.8)
Wasting (WLZ < −2)	0/36 (0)

**Table 3:** Pearl millet-based food product acceptability among children and mothers.

Recipe		Consumption (g) by Children			Overall Hedonic Score from mothers		
No.	Sweet food product	FeZnPM mean ± SD	CPM mean ± SD	*P*-value	FeZnPM mean ± SD	CPM mean ± SD	*P*-value
1	Cookies	18.71 ± 11.63	29.53 ± 14.25	0.002	8.09 ± 0.46	8.48 ± 0.51	0.002
2	Peanut *laddu*	24.33 ± 5.32	34.49 ± 17.59	0.005	7.28 ± 0.70	8.33 ± 0.48	<0.0001
3	*Sheera*	24.03 ± 11.37	22.50 ± 8.42	0.51	8.29 ± 0.59	8.24 ± 0.44	0.72
4	*Churma laddu*	31.49 ± 16.29	30.16 ± 11.76	0.70	8.03 ± 0.59	8.37 ± 0.49	0.01
5	Cake	18.94 ± 8.07	18.36 ± 8.05	0.78	8.39 ± 0.64	8.57 ± 0.50	0.22
6	*Nankhatai*	27.20 ± 12.90	19.27 ± 6.57	0.002	7.94 ± 0.61	8.32 ± 0.60	0.01
7	*Porridge*	21.62 ± 7.78	16.11 ± 7.85	0.01	7.55 ± 0.56	7.64 ± 0.49	0.47
8	*Puranpoli*	24.94 ± 8.09	27.13 ± 13.89	0.52	7.97 ± 0.40	7.55 ± 0.51	0.0006
	**Savory food product**						
9	*Khichdi*	9.82 ± 3.91	16.59 ± 6.87	<0.0001	8.29 ± 0.52	8.34 ± 0.48	0.69
10	*Upma*	9.30 ± 5.23	21.87 ± 9.51	<0.0001	8.29 ± 0.62	8.23 ± 0.43	0.64
11	*Dhokla*	28.14 ± 13.06	15.74 ± 8.60	<0.0001	8.06 ± 0.56	8.33 ± 0.48	0.04
12	*Idli*	21.34 ± 10.35	15.62 ± 6.49	0.009	8.15 ± 0.44	8.28 ± 0.45	0.26
13	Vegetable cutlet	53.16 ± 28.43	24.60 ± 10.58	<0.0001	8.21 ± 0.55	8.26 ± 0.45	0.66
14	*Kothimbir wadi*	26.77 ± 9.49	15.29 ± 5.30	0.42	7.85 ± 0.44	8.36 ± 0.49	<0.0001
15	*Thepla*	15.87 ± 9.56	21.32 ± 8.06	0.0001	7.97 ± 0.47	8.43 ± 0.57	0.0007
16	*Pav* (40%) *Bhaji* (51%)	27.40 ± 9.89	15.18 ± 6.49	0.16	7.18 ± 0.64	7.79 ± 0.41	<0.0001
	*Pav*	14.36 ± 7.98	9.29 ± 3.97	0.002	7.47 ± 0.51	8.29 ± 0.46	<0.0001
	*Bhaji*^a^	13.06 ± 7.98	31.11 ± 9.92	0.002			
17	*Pakoda*	24.60 ± 10.58	25.11 ± 13.90	0.0002	8.03 ± 0.59	8.15 ± 0.36	0.31
18	*Vada*^b^	19.19 ± 5.84	30.80 ± 12.13	0.01		8.30 ± 0.46	n/a

*Bold font indicates statistically significant values*.

*^a^Data for biofortified and conventional “bhaji” hedonic scores missing*.

*^b^Data for biofortified “Vada” hedonic scores missing*.

Mothers gave higher hedonic scores to foods made with CPM (“Overall” mean hedonic score: 8.22) compared to FeZnPM (“Overall” mean hedonic score: 7.95; *P* = 0.01) but had no preference or preferred FeZnPM in terms of color, taste, and overall score in at least 50% of recipes (Tables 3–6). In contrast, mothers tended to give higher ratings to CPM in terms of Odor (Table 5). For specific food items, Mothers gave the highest hedonic ratings to *churma laddu, upma*, and *nankhatai* (Table 3).

**Table 4:** Mothers’ color acceptability ratings: mean hedonic scores.

Recipe	FeZnPM		CPM		*P*-value	Higher rating
	*N*	Color mean ± SD	*N*	Color mean ± SD		
1. Cookies	33	7.64 ± 0.60	31	8.00 ± 0.37	0.005	CPM
2. Peanut *laddu*	36	6.64 ± 0.87	36	7.94 ± 0.23	<0.0001	CPM
3. *Sheera*	32	6.22 ± 0.91	30	8.00 ± 0.00	<0.0001	Conventional
4. *Churma laddu*	36	7.75 ± 0.77	30	8.00 ± 0.26	0.07	Tie
5. Cake	33	7.97 ± 0.39	31	7.74 ± 0.44	0.03	FeZnPM
6. *Nankhatai*	35	8.26 ± 0.44	31	7.90 ± 0.40	0.00	FeZnPM
7. Porridge	33	8.03 ± 0.73	34	7.94 ± 0.24	0.51	Tie
8. *Puranpoli*	34	7.62 ± 0.70	29	8.21 ± 0.41	0.00	CPM
9. *Khichdi*	17	7.94 ± 0.56	33	7.88 ± 0.33	0.67	Tie
10. *Upma*	34	8.18 ± 0.72	32	8.00 ± 0.00	0.16	Tie
11. *Dhokla*	32	7.47 ± 0.67	31	7.58 ± 0.50	0.45	Tie
12. *Idli*	33	7.48 ± 0.62	28	7.39 ± 0.50	0.52	Tie
13. Vegetable cutlet	33	7.88 ± 0.60	30	7.77 ± 0.50	0.42	Tie
14. *Kothimbirwadi*	33	7.67 ± 0.65	28	8.50 ± 0.51	<0.0001	CPM
15. *Thepla*	33	7.73 ± 0.80	30	7.97 ± 0.49	0.15	Tie
16a. *Pav*	33	6.67 ± 0.60	29	7.62 ± 0.49	<0.0001	CPM
16b. *Bhaji*	30	7.13 ± 0.57	28	8.76 ± 0.44	<0.0001	CPM
17. *Pakoda*	33	7.76 ± 0.50	33	7.97 ± 0.30	0.04	CPM
18. *Vada*^a^		37	7.76 ± 0.43	n/a	n/a

*^a^Data for biofortified “Vada” missing*.

**Table 5:** Mothers’ odor acceptability ratings: mean hedonic scores.

Recipe	FeZnPM		CPM		*P*-value odor	Higher rating
	*N*	Odor mean ± SD	*N*	Odor mean ± SD		
1. Cookies	33	7.55 ± 0.56	31	8.13 ± 0.34	<0.0001	CPM
2. Peanut *laddu*	36	6.33 ± 1.12	36	7.67 ± 0.59	<0.0001	CPM
3. *Sheera*	32	6.44 ± 0.76	30	8.50 ± 0.68	<0.0001	CPM
4. *Churma laddu*	36	7.47 ± 0.65	30	8.53 ± 0.51	<0.0001	CPM
5. Cake	33	6.91 ± 0.77	31	7.65 ± 0.49	<0.0001	CPM
6. *Nankhatai*	35	7.77 ± 0.69	31	7.74 ± 0.51	0.84	Tie
7. Porridge	33	7.82 ± 0.58	34	7.53 ± 0.56	0.04	FeZnPM
8. *Puranpoli*	34	6.68 ± 0.81	29	8.24 ± 0.58	<0.0001	CPM
9. *Khichdi*	17	7.71 ± 0.77	33	7.79 ± 0.55	0.70	Tie
10. *Upma*	34	7.56 ± 0.61	32	8.25 ± 0.51	<0.0001	CPM
11. *Dhokla*	32	6.78 ± 0.75	31	7.35 ± 0.49	<0.007	CPM
12. *Idli*	33	6.70 ± 0.73	28	7.54 ± 0.51	<0.0001	CPM
13. Vegetable cutlet	33	6.76 ± 0.61	30	7.70 ± 0.60	<0.0001	CPM
14. *Kothimbirwadi*	33	6.42 ± 0.56	28	8.36 ± 0.49	<0.0001	CPM
15. *Thepla*	33	7.70 ± 0.59	30	8.20 ± 0.71	0.004	CPM
16a. *Pav*	33	6.42 ± 0.79	29	7.38 ± 0.49	<0.0001	CPM
16b. *Bhaji*	30	6.63 ± 0.81	28	8.14 ± 0.52	<0.0001	CPM
17. *Pakoda*	33	7.12 ± 0.78	33	7.73 ± 0.67	0.001	CPM
18. *Vada*^a^			37	7.32 ± 0.53	n/a	n/a

*^a^Data for biofortified “Vada” missing*.

**Table 6:** Mothers’ taste acceptability ratings: mean hedonic scores.

Recipe	FeZnPM		CPM		*P*-value taste	Higher rating
	*N*	Taste Mean ± SD	*N*	Taste Mean ± SD		
1. Cookies	33	8.06 ± 0.50	31	8.68 ± 0.48	<0.0001	CPM
2. Peanut *laddu*	36	7.33 ± 0.68	36	8.67 ± 0.48	<0.0001	CPM
3. *Sheera*	32	7.94 ± 0.44	30	8.17 ± 0.38	0.03	FeZnPM
4. *Churma laddu*	36	8.31 ± 0.67	30	8.83 ± 0.38	0.0002	CPM
5. Cake	33	7.91 ± 0.77	31	8.23 ± 0.50	0.05	Tie
6. *Nankhatai*	35	8.29 ± 0.67	31	8.32 ± 0.65	0.82	Tie
7. Porridge	33	8.30 ± 0.68	34	8.24 ± 0.61	0.67	Tie
8. *Puranpoli*	34	8.24 ± 0.50	29	8.34 ± 0.55	0.41	Tie
9. *Khichdi*	17	8.29 ± 0.59	33	8.33 ± 0.60	0.83	Tie
10. *Upma*	34	8.35 ± 0.54	32	8.69 ± 0.47	0.01	CPM
11. *Dhokla*	32	8.25 ± 0.51	31	7.58 ± 0.50	<0.0001	FeZnPM
12. *Idli*	33	7.88 ± 0.70	28	7.43 ± 0.50	0.005	FeZnPM
13. Vegetable cutlet	33	8.27 ± 0.45	30	8.23 ± 0.43	0.72	Tie
14. *Kothimbirwadi*	33	7.85 ± 0.51	28	8.29 ± 0.46	0.0008	CPM
15. *Thepla*	33	7.97 ± 0.47	30	8.17 ± 0.38	0.07	Tie
16a. *Pav*	33	7.21 ± 0.65	29	7.52 ± 0.51	0.04	CPM
16b. *Bhaji*	30	7.47 ± 0.57	28	8.43 ± 0.50	<0.0001	CPM
17. *Pakoda*	33	8.09 ± 0.29	33	8.24 ± 0.56	0.17	Tie
18. *Vada*^a^			37	8.19 ± 0.62	n/a	n/a

*^a^Data for biofortified “Vada” missing*.

### Anthropometry

Data for sex and age were missing from two participants; therefore, anthropometric *z*-scores were calculated for the remaining sample of *n* = 36. On average, mean anthropometric *z*-scores were below the reference standard for weight-for-length, length-for-age, or weight-for-age (Table 2). Stunting (LAZ < −2) affected nearly half of the participants, while 27% of participants were underweight (WAZ < −2).

## Discussion

The present study is the first to develop several pearl millet-based complementary foods and to assess the acceptability of these food products among infants living in the urban slums of Mumbai, India. The acceptability of complementary food products was primarily determined by each child’s 3-day mean food intake along with mothers’ hedonic scores. Complementary food products developed from both varieties of pearl millet were highly accepted by children and their mothers.

There are limited studies on the acceptability of biofortified pearl millet-based complementary foods among young children (22). A recently published randomized controlled trial from our research group studied the effect of consuming biofortified pearl millet on iron status indicators compared to conventional pearl millet in school children from Maharashtra, India (10). These school children regularly consumed pearl millet-based unleavened bread (*bhakri*) for 6 months. Similar to our findings, no significant differences were observed between the consumption of biofortified and conventional pearl millet, and consumption of *bhakri* was high, indicating the high acceptance by school children (10). In a hospital-based feeding trial from southern India, the investigators studied the absorption of iron and zinc from biofortified pearl millet-based complementary foods (*sheera, upma*, and *roti*) compared to the same food products prepared using conventional pearl millet in young children (22–35 months of age) (16, 23). Children consumed an average of ~60 g pearl millet flour per day, again indicating the acceptance of pearl millet-based food products in 22- to 35-month-old children. Acceptability of other biofortified crops such as cassava has also been tested in young children and their caregivers (22). An acceptability study from Kenya compared the sensory acceptability of the consumption of foods prepared using biofortified cassava vs. white cassava in school children (7–12 years of age) and their caregivers (24). This study demonstrated that the biofortified cassava was well-accepted by the children and their caregivers compared to white cassava (24).

Consistent with our results, high acceptability of cereal and lipid-based complementary foods by toddlers was observed in other feeding studies from Peru (25) and Ghana (26). In our study, though the overall hedonic score responses from the mothers showed that CPM was preferred to FeZnPM, the quantitative data demonstrate that the consumption and acceptability of complementary foods prepared with both FeZnPM and CPM were equally high in this population. Similar hedonic score responses were observed in other studies from Peru (25) and Ghana (26) indicating that mothers’ responses can be biased, and direct measure of food consumption by the toddlers are more conclusive evidence of food acceptability, as reported in Bangladesh (27).

We observed variability in consumption volume among children, which may be attributed to the age range included (12–18 months) wherein some children are accustomed to consuming solid food (thereby eating more) while others are just beginning complementary feeding (who may still be breastfeeding, limiting their capacity for the pearl millet). This variability may be minimized by increasing the sample size in future acceptability studies, or excluding children who have already started complementary feeding.

In the preliminary phase of acceptability study, we observed some limitations including mothers’ refusal to feed their children in the presence of other caregivers or in a group due to presence of existing socio-cultural myths and taboos. Another important limitation we observed was that some mothers fed their child at home before bringing them to the feeding center as the child were reported to be very hungry. If the child refused to eat the study food product, mothers tended to breastfeed them which may have influenced their child’s hunger levels and consumption of the study food products.

In this study, we determined which biofortified pearl millet food products are the most accepted and would, therefore, have a higher likelihood of being consumed as part of the daily diet among young children. For example, *churma laddu* (described in Table 1) made with FeZnPM variety ICTP-8203 was most accepted by both mothers and infants. This indicates that similar recipes would be ideal candidates for inclusion in the proposed randomized controlled feeding trial (ClinicalTrials.gov ID: NCT02233764; Clinical Trials Registry of India (CTRI), reference number REF/2014/10/007731, CTRI number CTRI/2015/11/006376) to test the efficacy of consuming iron- and zinc-biofortified pearl millet like ICTP-8203 in improving iron status, growth, immune function, and cognition among young children.

## Ethics Statement

This study was carried out in accordance with the recommendations of Intersystem Biomedica Ethics Committee (ISBEC) (Ethic Committee Registration No. ECR/108/Indt/MH/2013) with written informed consent from all participants. All participants gave written informed consent in accordance with the Declaration of Helsinki. The protocol was approved by the Intersystem Biomedica Ethics Committee (ISBEC).

## Author Contributions

SM, JH, JF, SU, PG, and AK contributed to the concept and design of the work; VT, AS, and AT contributed to acquisition of data for the work; SH analyzed the data; SH, SV, SM, JH, JF, SU, PG, and AK interpreted the data; SH and SV wrote the initial draft of the manuscript; all authors contributed to critical revisions of the work; all authors gave final approval of the version to be published, SM had primary responsibility for the final content; all authors are in agreement that they will be accountable for all aspects of the work in ensuring that questions related to the accuracy or integrity of any part of the work are appropriately investigated and resolved.

## Conflict of Interest Statement

SM is an unpaid board member for a diagnostic start up focused on developing point-of-care assays for nutritional status informed by his research as a faculty member at Cornell University. All other authors declare that the research was conducted in the absence of any commercial or financial relationships that could be construed as a potential conflict of interest.
