# Corrigendum: Acceptability of Iron- and Zinc-Biofortified Pearl Millet (ICTP-8203)-Based Complementary Foods among Children in an Urban Slum of Mumbai, India

**DOI:** 10.3389/fnut.2018.00092

**Published:** 2018-10-18

**Authors:** Samantha Lee Huey, Sudha Venkatramanan, Shobha A. Udipi, Julia Leigh Finkelstein, Padmini Ghugre, Jere Douglas Haas, Varsha Thakker, Aparna Thorat, Ashwini Salvi, Anura V. Kurpad, Saurabh Mehta

**Affiliations:** ^1^Division of Nutritional Sciences, Cornell University, Ithaca, NY, United States; ^2^Department of Food Science and Nutrition, SNDT Women's University, Mumbai, India; ^3^St. John's Research Institute, Division of Nutrition, Bangalore, India; ^4^Institute for Nutritional Sciences, Global Health, and Technology (INSiGHT), Cornell University, Ithaca, NY, United States

**Keywords:** pearl millet, Dhanashakti, iron, iron deficiency, biofortification, acceptability, children

In the original article, there was an error in affiliation 3. The correct affiliation is “St. John's Research Institute, Division of Nutrition, Bangalore, India.”

In the original article, there was an error. The name of the biofortified pearl millet, referenced as **ICTP-8203** in the original article, is actually the name of the non-biofortified varietal (the iron-biofortified varietal is named **ICTP-8203Fe**). The name ICTP-8203 is corrected to Dhanashakti (or FeZnPM) to avoid confusion between these varietal names, since the name of the iron-biofortified pearl millet has been Dhanashakti since 2014 (the year of its official release).

A correction has been made to the title. The correct title is “Acceptability of Iron- and Zinc-Biofortified Pearl Millet (Dhanashakti)-Based Complementary Foods among Children in an Urban Slum of Mumbai, India.”

A correction has been made to the keywords. The correct keywords are: pearl millet, Dhanashakti, iron, iron deficiency, biofortification, acceptability, children

A correction has been made to the Abstract, Introduction, Materials and Methods, and Discussion. The corrected paragraphs appear below.

## Abstract

Biofortification, a method for increasing micronutrient content of staple crops, is a promising strategy for combating major global health problems, such as iron and zinc deficiency. We examined the acceptability of recipes prepared using iron- and zinc-biofortified pearl millet (FeZnPM) (~80 ppm Fe, ~34 ppm Zn, varietal Dhanashakti), compared to conventional pearl millet (CPM) (~20 ppm Fe, ~19 ppm Zn) in preparation for an efficacy trial. Our objective was to examine the acceptability of FeZnPM compared to CPM among young children and mothers living in the urban slums of Mumbai. Standardized traditional feeding program recipes (*n* = 18) were prepared with either FeZnPM or CPM flour. The weight (g) of each food product was measured before and after consumption by children (*n* = 125) and the average grams consumed over a 3-day period were recorded. Mothers (*n* = 60) rated recipes using a 9-point hedonic scale. Mean intakes and hedonic scores of each food product were compared using *t*-tests across the two types of pearl millet. There were no statistically significant differences in consumption by children (FeZnPM: 25.27 ± 13.0 g; CPM: 21.72 ± 6.90 g) across the food products (*P* = 0.28). Overall mean hedonic scores for all recipes were between 7 and 9 points. CPM products were rated higher overall (8.22 ± 0.28) compared to FeZnPM products (7.95 ± 0.35) (*P* = 0.01). FeZnPM and CPM were similarly consumed and had high hedonic scores, demonstrating high acceptability in this population. These results support using these varieties of pearl millet in a proposed trial [http://Clinicaltrials.gov ID: NCT02233764; Clinical Trials Registry of India (CTRI), reference number REF/2014/10/007731, CTRI number CTRI/2015/11/006376] testing the efficacy of FeZnPM for improving iron status and growth.

## Introduction, paragraph number 3

A locally grown variety of iron- and zinc-biofortified pearl millet (Dhanashakti or FeZnPM) with four times higher iron concentration than conventional pearl millet (CPM) has the potential to improve iron status in these populations (10, 14). Traditionally, pearl millet is ground into flour, roasted, and consumed in the form of non-leavened breads called bhakri or roti as part of the daily diet. Dhanashakti, shown to have comparable if not higher yield than conventional varieties (15), has demonstrated efficacy in improving iron status in older children who consumed bhakri twice daily for 6 months (10, 16). However, flatbreads like bhakri are not ideal weaning or complementary foods for young children and infants to consume due to their tougher and chewier texture. The main objective of this study was to formulate and test the acceptability (in terms of volume consumed and sensory characteristics) of new pearl millet-based palatable complementary food products for weaning infants. The food products with highest acceptability would be ideal candidates for a randomized controlled trial testing the efficacy of biofortified pearl millet for improving iron status in infants and young children.

## Materials and methods, intervention, paragraph number 1

Pearl millets CPM and FeZnPM were transported from Maharashtra and Gujarat, respectively, in 50-kg gunny bags and stored in a climate-controlled space (humidity level <50%, 25°C).

## Discussion, paragraph number 7

In this study, we determined which biofortified pearl millet food products are the most accepted and would, therefore, have a higher likelihood of being consumed as part of the daily diet among young children. For example, churma laddu (described in Table 1) made with FeZnPM variety Dhanashakti was most accepted by both mothers and infants. This indicates that similar recipes would be ideal candidates for inclusion in the proposed randomized controlled feeding trial (ClinicalTrials. Gov ID: NCT02233764; Clinical Trials Registry of India (CTRI), reference number REF/2014/10/007731, CTRI number CTRI/2015/11/006376) to test the efficacy of consuming iron- and zinc-biofortified pearl millet like Dhanashakti in improving iron status, growth, immune function, and cognition among young children.

The authors apologize for these errors and state that this does not change the scientific conclusions of the article in any way.

### Conflict of interest statement

SM is an unpaid board member for a diagnostic start up focused on developing point-of-care assays for nutritional status informed by his research as a faculty member at Cornell University. The remaining authors declare that the research was conducted in the absence of any commercial or financial relationships that could be construed as a potential conflict of interest.
